# Medication Regimen Complexity and Patient-Centred Outcomes in Patients Undergoing Peritoneal Dialysis

**DOI:** 10.3390/healthcare12212121

**Published:** 2024-10-24

**Authors:** Jing Xin Goh, Kamal Sud, Wubshet Tesfaye, Connie Van, Shrey Seth, Surjit Tarafdar, Ronald L. Castelino

**Affiliations:** 1 School of Pharmacy, Faculty of Medicine and Health, The University of Sydney, Sydney, NSW 2006, Australiaronald.castelino@sydney.edu.au (R.L.C.); 2 Sydney Medical School, Faculty of Medicine and Health, The University of Sydney, Sydney, NSW 2006, Australia; 3 Nepean Kidney Research Centre, Department of Renal Medicine, Nepean Hospital, Kingswood, NSW 2747, Australia; 4 School of Pharmacy, Faculty of Health and Behavioural Sciences, The University of Queensland, St Lucia, Brisbane, QLD 4072, Australia; 5 Faculty of Medicine, Western Sydney University, Sydney, NSW 2751, Australia; 6 Department of Medicine, Blacktown Hospital, Western Sydney Local Health District, Blacktown, NSW 2148, Australia; 7 Pharmacy Department, Blacktown Hospital, Western Sydney Local Health District, Blacktown, NSW 2148, Australia

**Keywords:** kidney failure, peritoneal dialysis, medication regimen complexity index, adherence, health-related quality of life

## Abstract

Background: Although patients undergoing peritoneal dialysis (PD) typically have complex treatment needs, the effect of medication regimen complexity on patient outcomes has not been thoroughly evaluated. This study aims to quantify medication regimen complexity and evaluate patient-centred outcomes including medication adherence and its determinants in patients undergoing PD. Methods: This study combined a retrospective audit of baseline data with a prospective evaluation of patient-related outcomes among patients undergoing PD at a large metropolitan dialysis centre in Australia. Medication regimen complexity was assessed using the 65-item Medication Regimen Complexity Index (MRCI), while patient outcomes were evaluated with validated self-reported questionnaires, including the 4-item Morisky–Green–Levine Scale (MGLS), EQ-5D-5L and EQ VAS. Results: A total of 131 patients participated [median age 67 (IQR 57–74) years]. Patients on PD were found to have complex medication regimens with an average MRCI score of 28.6 ± 11.4. Over half of the participants were deemed to be adherent to their prescribed medications as measured by the MGLS (*n* = 79; 60.3%). Male participants were more likely to be non-adherent to medications compared to female participants (OR 2.465; 95% CI 1.055–5.759). Participants with higher serum phosphate levels were 2.5 times more likely to report non-adherence to their medications (OR 2.523; 95% CI 1.247–5.104), while a higher health-related quality of life (HRQoL) was associated with medication adherence (OR 0.151, 95% CI 0.031–0.732). Conclusions: Patients on PD are prescribed complex medication regimens in addition to PD treatments that they perform at home. Patients on PD who were adherent to their medications had significantly better outcomes in terms of HRQoL and serum phosphate levels compared to non-adherent patients.

## 1. Introduction

Peritoneal dialysis (PD) is the preferred form of kidney replacement therapy for many people with kidney failure, as it is delivered at home. Several countries have adopted a ‘PD-first’ or ‘PD-favoured’ approach because PD requires less personnel and infrastructure [1]. According to the latest data from the Australia and New Zealand Dialysis and Transplant Registry, there were 2657 (17%) PD patients in Australia, of which the majority (73%) were receiving automated peritoneal dialysis (APD) in 2022 [2].

It has been suggested that PD confers several advantages over haemodialysis (HD) in terms of preserving residual kidney function, as well as providing a better health-related quality of life (HRQoL) [3]. Nevertheless, regardless of the dialysis treatment modality, polypharmacy is an issue in patients with kidney failure since patients invariably require multiple medications to alleviate the symptoms associated with kidney failure and to manage multiple comorbidities and complications such as hypertension, cardiovascular diseases, bone mineral diseases and anaemia [4]. Recent studies have shown that patients undergoing PD treatment have a similar medication burden to those receiving facility-based HD treatments [5,6]. As most of the evidence to date has focused on patients on HD, with limited studies on patients undergoing PD, little is known about the medication regimen complexity, medication adherence and patient-centred outcomes in this population with kidney failure. Thus, this study aims to (i) quantify the level of medication regimen complexity in people undergoing PD; (ii) evaluate patient-centred outcomes including HRQoL and the incidence of hospitalisation(s) over the preceding 12-month period in people on PD; and (iii) compare differences in self-reported patient-centred outcomes among PD patients based on their medication adherence.

## 2. Materials and Methods

### 2.1. Study Design, Setting and Population

This was a retrospective audit of relevant clinical information, and a prospective evaluation of patient-related outcomes among patients undergoing PD recruited from a large metropolitan dialysis centre in New South Wales, Australia. The PD service provides care and patient training for approximately 325 patients on PD. Patients were approached for participation in the study between March 2024 and May 2024. We excluded patients who were temporarily undergoing HD, and those receiving kidney-supportive care/end of life care while on PD. Patients who had a language barrier or cognitive impairment were also excluded from the study. After informed consent, participants’ characteristics—age, gender, primary language, marital status, ethnic group, smoking status, alcohol consumption, laboratory values, comorbidities and medications—were obtained from the patients’ electronic medical records, while HRQoL and medication intake behaviour were obtained via phone interviews or during their follow up appointment at the PD clinic. Complexity of prescribed PD regimens or adherence of PD treatments at home were not assessed. This study was approved by the Institutional Human Research Ethics Committee (2019/PID03039).

### 2.2. Measures

#### 2.2.1. Variables

A list of the participants’ medications was obtained from the electronic medical record (eMR). The accuracy of these medication lists was verified with participants during the interview and/or correlated with dispensing history provided by their community pharmacy, if required. The medication list included both prescription and over-the-counter medications. Medications such as antibiotics for short-term use and herbal supplements were excluded from the medication list. If necessary, to resolve any discrepancies between participants’ medication lists and the eMR or pharmacy’s dispensing history, a discussion between the renal pharmacist, research pharmacist and the patient was conducted.

The Medication Regimen Complexity Index (MRCI) [7] and daily pill burden assessment were used to quantify medication regimen complexity. The MRCI is a 65-item tool developed to evaluate the complexity of a patient’s drug therapy by considering three different components: (i) dosage form, (ii) dosing frequency and (iii) additional directions concerning administration. Based on a weighted scoring system, each medication was scored on the three different components, and the individual component scores were then summed to create a total score for medication complexity. A base score of 1 was given to (each) a tablet/capsule dosage form and a once daily dosing frequency. There was no established maximum score as the total score increases in line with the quantity of medications and the increase in regimen complexity. An electronic data capture and coding tool developed by Libby et al. was used to compute the total MRCI scores for each participant [8]. Pill burden was determined as the total number of medications taken orally by the participants each day. For medications that were not taken daily, i.e., once or several times a week, the total pill burden for a week was divided by 7. Medications administered on an “as needed” basis and non-orally administered medications such as topical, injectable and inhaled formulations were excluded from the pill burden count. Medications were classified as per the World Health Organisation Anatomical Therapeutic Chemical (ATC) Classification system.

#### 2.2.2. Outcomes

Self-reported medication adherence was evaluated using the 4-item Morisky–Green–Levine Scale (MGLS) which consists of four questions with binary (yes/no) response options [9]. It is a validated, reliable and widely used instrument in the clinical setting for different chronic illnesses including chronic kidney disease (CKD) [10,11,12,13,14]. Participants were classified as non-adherent if they responded ‘yes’ to any of the four questions. An objective assessment of a random serum phosphate level close to the interview date was also carried out to further confirm medication adherence.

EQ-5D-5L and EQ VAS developed by EuroQol were used to measure HRQoL (registration ID: 61889) [15]. EQ-5D-5L comprises five dimensions—mobility, self-care, usual activities, pain/discomfort and anxiety/depression. Each dimension has 5 response levels of perceived problems, starting with level 1 indicating no problem, to level 5 indicating extreme problems. Each dimension represents a standard index score ranging from −0.3 to 1, where 1 reflects the most optimal state of health. EQ VAS forms part of the EQ-5D-5L that served as a quantitative assessment of participants’ overall health status. It is a visual analogue scale on which participants are required to rate their general health from 0 to 100.

The incidence of hospitalisation(s) 12 months prior to interview was obtained from electronic medical records. Only unplanned admissions were included in the study data, while admissions resulting in an <24 h stay in the hospital and elective admissions were excluded.

### 2.3. Statistical Analyses

Statistical analysis was performed using the Statistical Package for the Social Sciences (SPSS version 29.0.1.0, Armonk, NY, USA: IBM Corp). All study data were tested for normality using the Shapiro–Wilk test. Continuous variables that were normally distributed were presented as the mean ± standard deviation (SD), whereas non-normally distributed variables were presented as the median (interquartile range [IQR]) (25–75%). Frequency (percentage) was used to report proportions for categorical variables.

Participant characteristics such as sociodemographic and medical profile, medication regimen complexity, number of medications, pill burden and outcomes (HRQoL, serum phosphate level, incidence and number of hospitalisation(s)) were compared between adherent and non-adherent groups of participants. Student’s *t*-test was used to compare the means of normally distributed continuous variables, while the comparison between nonnormally distributed variables were performed using the Mann–Whitney U test. Categorical variables were compared using the Chi-square test. Study variables with *p* values < 0.1 for the univariate analyses (gender, serum phosphate level, EQ-5D-5L index and EQ VAS) as well as the MRCI were entered into the binary logistic regression model to identify predictors of medication non-adherence. Effect sizes were reported using odds ratios (ORs) and 95% confidence intervals (CIs). A two-sided *p*-value of <0.05 was considered statistically significant.

## 3. Results

Overall, 131 [71 automated peritoneal dialysis (APD) and 60 continuous ambulatory peritoneal dialysis (CAPD)] patients consented to the study (response rate 65.5%). More than half of the patients were deemed adherent to their prescribed medications as assessed by the MGLS (*n* = 79; 60.3%). The baseline sociodemographic and medical characteristics of study participants are reported in Table 1.

### 3.1. Characteristics of Study Participants

The study participants were predominantly men (*n* = 85; 64.9%), with a median age of 67 (IQR 57–74) years. More than half of the participants were treated with the APD modality (*n* = 71; 54.2%). There was no significant difference in adherence between the different PD modalities. There were also no significant differences in sociodemographic characteristics such as marital status, level of education, alcohol consumption and smoking, as well as laboratory parameters (haemoglobin and ferritin levels), between the adherent and non-adherent groups of participants. In general, the study participants had a relatively high comorbidity burden, with a median of six (IQR 5–8) comorbid conditions. The most common comorbidities were hypertension (*n* = 123; 93.9%), followed by diabetes mellitus (*n* = 75; 57.3%), and atherosclerotic disease (*n* = 47; 35.9%).

Participants were prescribed a median of 11 (IQR 8–14) medications, with a daily pill burden of 14.5 (IQR 10.0–21.0). Only four (3.1%) participants were prescribed fewer than five medications, indicating that the vast majority of participants (96.9%) experienced polypharmacy (use of ≥5 medications). In line with the common comorbidities present in this cohort of participants, we identified that the most prescribed drug class was from the cardiovascular system pharmacological group (31.4%), followed by the alimentary tract and metabolism (31.0%), and the blood and blood-forming organs (13.2%) group of medications. The mean MRCI score of the study population was 28.6 (SD 11.4), with the dosing frequency component contributing the most to the total score. The number of medications prescribed, daily pill burden, total MRCI and individual MRCI component scores did not significantly differ between the adherent and non-adherent participants.

### 3.2. Outcomes and Determinants of Medication Non-Adherence

Table 2 presents the patient-centred outcomes of the study participants. Of note, participants who were adherent to their prescribed medications had a higher EQ-5D-5L score compared to their non-adherent counterparts [0.926 (IQR 0.755–0.968) vs. 0.734 (IQR 0.333–0.928), respectively; *p* = 0.002]. Similarly, the EQ VAS score was significantly higher among those who were adherent compared to the non-adherent group of participants [70 (IQR 50–80) vs. 50 (IQR 40–80), respectively; *p* = 0.017]. Unsurprisingly, the objective assessment of serum phosphate level among adherent participants was shown to be lower compared to non-adherent participants [1.61 (IQR 1.36–2.06) vs. 1.80 (IQR 1.50–2.33), respectively; *p* = 0.019]. Nevertheless, there were no significant differences in the incidence and number of hospitalisation(s) 12 months prior to the date of interview between the two groups of participants.

When examining the predictors of medication non-adherence using the binary logistic regression model (Table 3), we found that male participants were more likely to be non-adherent to medications compared to female participants (OR 2.465; 95% CI 1.055–5.759; *p* = 0.037). Participants with a higher serum phosphate level were 2.5 times more likely to report non-adherence to their medications (OR 2.523; 95% CI 1.247–5.104; *p* = 0.010). On the other hand, a higher HRQoL was associated with medication adherence (OR 0.151, 95% CI 0.031–0.732; *p* = 0.019).

## 4. Discussion

This is the first study assessing medication regimen complexity, medication adherence and patient-centred outcomes focusing on patients receiving PD. We found that patients with kidney failure undergoing PD had complex medication regimens with an average MRCI score of 28.6 ± 11.4, which was notably higher than that reported for patients with pre-dialysis CKD (median MRCI 19.0) [12] as well as other chronic illnesses such as diabetes mellitus, HIV and psychiatric disorders (mean MRCI range 6.9–16.0) [16,17,18]. The MRCI scores of participants in this study were found to be comparable to studies involving patients undergoing facility-based [19,20] and home HD [21] treatments. Interestingly, at our dialysis centre, we found that the MRCI scores of participants undergoing PD were higher than those undergoing facility-based HD (median MRCI 20.8) (author’s unpublished study—currently under review). This may be due to a shift in the dose administration responsibility for several parenteral medications such as erythropoiesis-stimulating agent. The adherence rate among participants in this study (65.5%) was also shown to be slightly better compared to previous reports conducted among facility-based HD patients (≤50%) [19,21]. This could be attributed to several reasons as patients receiving PD are generally younger, more educated and have fewer comorbidities compared to patients treated with HD [22]. As a result, PD patients would be more independent in dealing with the challenges of managing their own treatment at home, hence their better motivation and commitment to their care plan. Nevertheless, the previous literature indicated the medication non-adherence rate in PD patients varied from 3.9% to 85% [23]. Differences in adherence assessment tools, patient demographics, healthcare systems and settings may contribute to such discrepancies.

Of note, the current study reported a significantly better HRQoL measured using the EQ-5D-5L index and EQ VAS among the adherent compared to the non-adherent group of participants. This finding is congruent with the findings of previous studies conducted among patients with pre-dialysis CKD [12] and in patients who underwent kidney transplantation [24]. In general, patients receiving PD have been shown to have a better QoL and other clinical outcomes compared to patients receiving HD [3,22], but a comparison of PD versus HD and the associated outcomes was not part of this study’s objectives. Compared to our study, which showed that male gender, serum phosphate levels and HRQoL were determinants of medication non-adherence, several other factors such as younger age and being from racial minority and lower socioeconomic groups were also identified as predictors of poorer medication adherence across all CKD stages [25]. Despite similar findings from a previous study suggesting poorer outcomes in males with CKD due to a lower adherence to treatment [26], the reasons for gender differences influencing medication adherence are multifactorial and could include sociodemographic, psychological and other factors. Furthermore, since a poor HRQoL has been well documented to be associated with increased morbidity and mortality, the enhancement of a patient’s HRQoL is increasingly important when monitoring the quality and effectiveness of kidney care, and is currently considered a high priority area in kidney research [27]. On another note, adherent participants in our study had lower random serum phosphate levels compared to non-adherent participants, indicating better phosphate control possibly due to a better phosphate binder and/or dietary adherence. This was also shown in previous studies conducted among patients undergoing HD [28,29]. Lower serum phosphate levels, in turn, were found to be a predictor of medication adherence in our study, further indicating the importance of the relationship between both outcomes in patients treated with PD. Since a limited amount of phosphate is removed through the peritoneal membrane, PD patients frequently develop hyperphosphatemia and are often prescribed phosphate binder medications to reduce dietary phosphate absorption [30]; phosphate binder adherence is crucial to reduce the risk of cardiovascular morbidity and mortality among PD patients.

The present study failed to show significant differences in measures of medication regimen complexity including the MRCI scores, number of medications prescribed and daily pill burden between the adherent and non-adherent groups of PD participants. Ghimire et al. and Parker et al. have also reported no association between medication complexity and adherence in both pre-dialytic and patients on dialysis [19,20], in contrast with other studies that indicated an inverse relationship between medication complexity and adherence in a similar patient group [13,31]. The lack of a standardised definition of medication regimen complexity would most likely account for the differences in findings between the studies. Nevertheless, the use of different medication regimen complexity assessment methods and MRCI as the main measure in our study, which includes comprehensive aspects of a medication regimen, enabled us to compare between the outcomes of different studies. Whilst there were no significant differences in the outcomes of hospital admission(s) one-year prior to baseline and the median number of hospitalisation(s) between the adherent and non-adherent groups of participants, the hospitalisation rate in PD participants in our study was found to be lower than that in a recent study comparing the hospitalisation rates between PD and HD patients [32].

This is the first study from Australia conducted among PD patients on medication regimen complexity and pill burden, as well as patient-centred outcomes. Additionally, in contrast to existing local literature [19] on dialysis patients featuring a smaller sample size (*n* < 60), this study was carried out in the largest PD centre in Australia with a sample size of *n* > 100. To obtain the best possible medication history and to minimise the potential inaccuracy of participants’ medication lists, we have obtained the lists of medications from several sources including the electronic medical record, verification with participants’ community pharmacy and confirmation during patient interview. Nonetheless, this study is not without limitations. Although this study was conducted in a large centre that caters to people from diverse background, this may not be representative of the entire Australian population. The use of a self-reported adherence tool may lead to an under-estimation of non-adherence due to recall and social desirability bias. However, the 4-item MGLS that was used in this study is commonly adopted in clinical practice due to its ease of implementation. Additionally, the use of serum phosphate level as an objective outcome measure may be influenced by other factors including dietary adherence, and hence may not accurately reflect patient outcomes related to medication complexity. Another caveat is that medication regimen complexity was only measured at a single timepoint, when participants were recruited into the study. This may not fully capture the complexity, potentially leading to an over- or under-estimation of medication regimen complexity as patients with kidney failure frequently have their treatment adjusted to manage multiple comorbidities which can cause dynamic changes in their health status. Such limitations of a cross-sectional study design could be addressed in future studies by incorporating a longitudinal study design to gain a better understanding of medication complexity among patients receiving PD. Finally, we have not assessed the impact of the complexity of prescribed PD regimens or adherence to PD treatments at home on patient-related outcomes as this was beyond the scope of our study.

## 5. Conclusions

Despite the complex medication regimens, PD patients who were adherent to their medications had significantly better patient-centred outcomes in terms of HRQoL and serum phosphate levels compared to non-adherent patients. This reinforces the importance of enhancing patient adherence as one of the main focuses in the practice of daily care for patients with kidney failure. Future research should consider interventional studies to address or improve medication adherence and regimen complexity among PD patients, and should recruit a larger sample of patients from multiple dialysis centres and various dialysis modalities.

## Figures and Tables

**Table 1:** Study characteristics based on participant self-reported medication adherence.

Variables	Total Cohort (*n* = 131)	Adherent (*n* = 79)	Non-Adherent (*n* = 52)	*p* Value
*Sociodemographic*				
**Median age, years (IQR)**	67 (57–74)	67 (58–74)	67 (54–74)	0.422
**APD modality, *n* (%)**	71 (54.2)	40 (50.6)	31 (59.6)	0.313
**Male gender, *n* (%)**	85 (64.9)	45 (57.0)	40 (76.9)	0.019 *
**Primary language, *n* (%)** **English** **Tagalog** **Mandarin**	63 (48.1) 16 (12.2) 8 (6.1)	38 (48.1) 9 (11.4) 4 (5.1)	25 (48.1) 7 (13.5) 4 (7.7)	
**Married/de facto, *n* (%)**	89 (67.9)	54 (68.4)	35 (67.3)	0.900
**Level of education (year 12 or less), *n* (%)**	71 (54.2)	41 (51.9)	30 (57.7)	0.515
*Clinical*				
**Total number of comorbid conditions, median (IQR)**	6 (5–8)	6 (5–8)	6 (5–9)	0.476
**Common comorbidities, *n* (%)** **Hypertension** **Diabetes mellitus** **Atherosclerotic disease** **Congestive heart failure** **Malignant neoplasm**	123 (93.9) 75 (57.3) 47 (35.9) 26 (19.8) 13 (9.9)	72 (91.1) 42 (53.2) 25 (31.6) 18 (22.8) 8 (10.1)	51 (98.1) 33 (63.5) 22 (42.3) 8 (15.4) 5 (9.6)	0.105 0.244 0.213 0.299 0.924
**Smoking (former and current), *n* (%)**	47 (35.9)	28 (35.4)	19 (36.5)	0.898
**Alcohol consumption (former and current), *n* (%)**	46 (35.1)	25 (31.6)	21 (40.4)	0.305
*Laboratory*				
**Haemoglobin (g/L), median (IQR)**	108 (98–117)	108 (99–117)	104 (96–117)	0.192
**Ferritin (µmol/L), median (IQR)**	290 (151–473)	295 (154–460)	278 (145–545)	0.976
*Medications*				
**No. of medications, median (IQR)** **≥5 medications, *n* (%)** **≥10 medications, *n* (%)**	11 (8–14) 127 (96.9) 87 (66.4)	11 (9–14) 76 (96.2) 56 (70.9)	11 (7–14) 51 (98.1) 31 (59.6)	0.549 0.542 0.181
**Patient prescribed with phosphate binder(s), *n* (%)**	100 (76.3)	57 (72.2)	43 (82.7)	0.165
**Prescribed medications by drug categories, *n* (%)** **Cardiovascular system** **Alimentary tract and metabolism** **Blood and blood forming organs**	460 (31.4) 455 (31.0) 194 (13.2)	268 (29.8) 281 (31.3) 118 (13.1)	192 (33.8) 174 (30.6) 76 (13.4)	
**Daily pill burden, median (IQR)**	14.5 (10.0–21.0)	15.0 (10.1–21.4)	14.2 (10.0–20.6)	0.358
**MRCI, mean (SD)** **A/dosage form** **B/dosing frequency** **C/additional directions**	28.6 (11.4) 4.9 (2.5) 17.2 (7.1) 6.6 (4.1)	28.7 (11.3) 4.9 (2.5) 17.8 (7.3) 6.1 (3.4)	28.6 (11.7) 4.9 (2.5) 16.4 (6.8) 7.4 (4.9)	0.962 0.958 0.279 0.093

Abbreviations: APD, automated peritoneal dialysis; IQR, interquartile range; MRCI, medication regimen complexity index; SD, standard deviation. * Statistical significance.

**Table 2:** Clinical and patient-related outcomes.

Variables	Total Cohort (*n* = 131)	Adherent (*n* = 79)	Non-Adherent (*n* = 52)	*p* Value
**EQ-5D-5L, median (IQR)**	0.889 (0.566–0.961)	0.926 (0.755–0.968)	0.734 (0.333–0.928)	0.002 *
**EQ VAS, median (IQR)**	60 (50–80)	70 (50–80)	50 (40–80)	0.017 *
**Serum phosphate level (mmol/L), median (IQR)**	1.69 (1.43–2.19)	1.61 (1.36–2.06)	1.80 (1.50–2.33)	0.019 *
**Hospital admission(s) 1 year prior to baseline, *n* (%)** **Number of hospital admission(s), median (IQR)**	65 (49.6) 0 (0–2)	37 (46.8) 0 (0–2)	28 (53.8) 1 (0–2)	0.432 0.409

Abbreviations: IQR, interquartile range; EQ-5D-5L, EuroQol-5 dimensions-5 level; EQ VAS, EuroQol visual analogue scale. * Statistical significance.

**Table 3:** Predictors of medication non-adherence using logistic regression analysis.

Variables	Adjusted OR (95% CI)	*p* Value
**Male gender**	2.465 (1.055–5.759)	0.037 *
**MRCI (total score)**	0.969 (0.933–1.007)	0.104
**Serum phosphate level, per 1 mmol/L**	2.523 (1.247–5.104)	0.010 *
**EQ-5D-5L**	0.151 (0.031–0.732)	0.019 *
**EQ VAS**	0.988 (0.967–1.011)	0.309

Abbreviations: EQ-5D-5L, EuroQol-5 dimensions-5 level; EQ VAS, EuroQol visual analogue scale; MRCI, medication regimen complexity index. * Statistical significance.

## Data Availability

The datasets generated and analysed are available upon request.
